# President’s Address—The Texas Surgical Society

**Published:** 1915-11

**Authors:** James E. Thompson

**Affiliations:** Galveston, Texas


### TEXAS MEDICAL JOURNAL

Dr. F. E. Daniel, Founder. Established July, 1885

MRS. F. E. DANIEL, - Publisher and Managing Editor

Published Monthly.—Subscription, $1.00 a Year.

VOL. XXXI AUSTIN, NOVEMBER, 1915 NO. 6

The publisher is not responsible for views of contributors

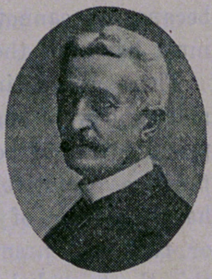

### Original Articles.

### President's Address—The Texas Surgical Society.

BY JAMES E. THOMPSON, M. D., GALVESTON, TEXAS.

*Gentlemen:*

When the charter members of the society paid me the great honor of electing me as its first president, I accepted the position with deep pleasure. I was gratified, first, by the compliment which was the outward manifestation of the esteem in which I was held by all of you, and, secondly, by the chance that the position afforded me to discuss in an authoritative manner some of the great ethical problems that are confronting us.

Population is increasing so fast in the State of Texas that a period of ten years transforms many towns so completely that they are unrecognizable from a business and social standpoint. Men spring up like mushrooms in every walk of life, and the cards are being shuffled so fast that a man who was without a peer in his business or profession ten years ago is now confronted with competitors who are as good or better than he. In our profession this is strikingly evident, because standards of medical education have been growing so steadily that man for man the graduate of ten years ago bears no comparison with the graduate of today, either in general culture or in special scientific knowledge.

Some of us and most of our predecessors received a scanty education in the medical schools, but climbed to knowledge afterwards in general practice. Amongst the older surgeons in the State there are few who learned their surgery in any other manner. Naturally endowed with good sense and judgment, they followed a surgical bias in their own practice until they gained proficiency. Reputations fully deserved, achieved under such conditions, excite our admiration. They also excite our sympathy, because we cannot help knowing that men of such mental ability must have felt their educational limitations very keenly.

There was a time in Texas when all the surgery was done by men who were engaged in general practice. I believe I have the proud honor of being the first surgeon in this State to limit his practice exclusively to surgery. At the present time there is hardly a town of over 20,000 inhabitants that does not have one or two men practicing surgery almost exclusively. In spite of this the overwhelming bulk of surgical work is being done by the general practitioner. Is this a proper state of affairs? Is it possible for a man practicing all branches in his profession to acquire sufficient skill to justify him in performing operations of a delicate and dangerous nature? In many instances the character of work done by general practitioners is extremely good and deserves the highest praise. But it is a deplorable fact that in the hands of the majority crude, sloppy work of an unscientific character is the rule. For one man fitted by nature and by education to practice surgery, there are a dozen who are unfitted in every way to deal with the practical side of the surgical art. How is it possible for a man busy day and night with the multitudinous duties of general practice to set apart special hours for his surgical work when his energies should be at his best? In what condition is he in the morning with serious operative problems to grapple with, after a night spent with some obstetric case? A weary man has no place in the operating room. An apparently simple case may bristle with complications, and in a moment a smooth, placid operation may be transformed into a desperate struggle to save life.

The American Surgical Association, which is a type of everything that is admirable in its contributions to the progress of surgery, practically limits its membership to men practicing surgery exclusively or teachers of surgery in reputable medical schools. Other surgical associations are not so exclusive, but admit men whose leanings are towards surgery and who have established a reputation in this branch. Every association in choosing between men of equal reputation gives the preference to surgical specialties, because it is a well recognized fact that in such a vast field work of greater value will be forthcoming from specialized effort.

Twenty charter members represented originally the available men in the State of Texas who were practicing surgery exclusively. The rest have got to be drawn from the ranks of the general practitioners. We have got magnificent material to draw from, and if new Fellows are chosen wisely we shall establish a society that will be the pride of its founders, and a leader in scientific progress. We shall reach this goal all the quicker if every new Fellow is urged to give to the practice of surgery his undivided efforts.

It is my opinion, and I believe I voice that held by the majority of the charter members, that this society ought to consist of surgeons who practice their specialty exclusively, and that we as a nucleus should impress upon all future Fellows the desirability of withdrawing from general practice at the earliest opportunity. Many aspirants to the Fellowship do so little general work that it would be a relief for them to discard it, and they would gladly do so if urged. Others would willingly take the step when membership in this society demands such a sacrifice.

If this society is to take a position of honor and dignity amongst the learned societies of the country, it must not hesitate to deal with any ethical problem that presents itself for consideration in a prompt, logical and dignified way. We must take our share in the great educational movements that are sweeping over the medical schools and hospitals of the country with irresistible force. We must add our weight and influence to that of the larger and more representative organizations who wield legislative powers. We should keep out of medical politics as generally understood, but we must be in full accord with all policies that lead to better scientific training and achievement.

During the last ten years remarkable changes have taken place in medical education, mainly due to the efforts of the Carnegie Committee and the Council of Medical Education of the American Medical Association. The result of the determined efforts of these two bodies to raise the standard of medical education and the plane of equipment of the medical schools, has been so successful that it has placed a large number of American schools in the proud position of being considered the best in the world, and has raised the vast majority to a place ranking equally with those of continental Europe. This change was not accomplished without opposition. The fight on the part of the proprietary schools was most bitter because they were threatened with absolute extinction. It is a matter of common history how some survived and others succumbed. Following in the wake of this movement, which has practically accomplished its purpose, another is about to be launched, which, if successful, will revolutionize the conduct and management of the larger hospitals of the country.

This is a scheme for standardization of the hospitals, by which they can be used as a means of educating medical students. It is proposed by the Council of Medical Education to lengthen the medical curriculum by one year, making it five years, and to insist on every graduate spending his fifth year as an interne in a well regulated hospital. A tour of the hospitals of the country will be made and their scientific and educational advantages analyzed, and the result will be published. If a hospital fails to meet reasonable requirements, service in its wards will not be accepted by the Council, and consequently no interne who accepts such a position will be able to count for time of residence as any part of his fifth year. The efforts of this will be to deprive every hospital badly managed or poorly equipped of internes, and they will be forced either to reform or to accept an inferior standing. The result will be that every large hospital will endeavor to meet the requirements in order to obtain internes.

What will these changes mean? In a hospital connected with a school of medicine the following will be the requirements: A sufficient number of beds under the exclusive control of teachers. A sufficient number of clinical teachers and assistants with continuous services. Proper equipment. Adequate services in all general and special branches of medicine and surgery. Sanitary wards. Satisfactory pathological laboratory and X-ray departments. Proper operating room facilities with equipment. Properly organized out-patient department. Obstetric service so organized that every candidate for graduation shall have seen a definite number of cases of labor. Lastly, a proper system of records, which shall include complete histories of both in-patients and out-patients.

In hospitals not connected with medical schools, the requirements will not be so rigid as to the number of assistants. The visiting staff must, however, be organized in such a manner that adequate services shall exist to permit of the rotation of the internes. The items as to the equipment and laboratory facilities will be demanded. The keeping of accurate system of records will be compulsory.

Not alone in Texas, but all over the United States, every town of a considerable size has in its midst one or more hospitals which are owned by business men or by charitable or by religious organizations. Will these institutions be affected by these proposed changes? To a slight degree we may expect improvement, but it will always be subservient to economic reform and monetary profit. If they improve in the direction of efficiency, it will not be as a rule for the sake of improvement, but because increased efficiency pays a dividend. As to improvement in organization, there is less hope because the majority of the men who use the institutions are interested only in the bodies and welfare of their patients, and not in the government of the institution. The visiting staff of some of these institutions consist of all the men who patronize it. In some there is no visiting staff, the doctors merely using the hospital as a nursing home. In others, again, there is an organization called a visiting staff, which rarely holds meetings and which possesses no power of legislation whatsoever. Usually no records are kept by the hospital authorities beyond those demanded by the health authorities of the city, which usually amounts to entering up in a book the name, age, sex, nativity, address and diagnosis. All these details are entered up by an interne who probably never sees the case. An operating book is kept, in which the name of the patient and the character of the operation are entered. This is often kept in a very imperfect and slovenly manner and entries are often made from hearsay. These institutions keep no permanent records. Most of the doctors write no histories. Even if they did, they would be burned with the sheets of the nurses' reports, which are never kept.

The most serious indictment of these institutions is their entire absence of any sense of responsibility as to their duties to society. They open their doors to any doctor and give him the privileges of working in their institution without any inquiries as to his efficiency. If he is morally unfit, as a rule, they find some means of refusing to receive his cases; but they take no notice of relative degrees of unfitness in the men who are using their institution. Any aspirant to fame as a surgeon can use their operating room, and command the services of their nurses, without a question being raised as to his fitness. Without question, they offer the protection of their institution to any man who sends them patients. It is impossible to help rumors of operating room tragedies from leaking out. Internes will talk, and occasionally one more courageous than another, will openly relate stories of incompetence and ignorance that are almost unbelievable. I believe that few of these institutions are guilty of allowing criminal practice to go on within their walls, at least, knowingly. But when no supervision is exercised, it is an easy matter for curettements to be practiced indiscriminately, presumably as a justifiable procedure, but in reality with a criminal intent. The abuse of the curette often goes hand in hand with an elastic conscience. What remedy can be offered to correct these abuses? First, I would suggest that each hospital appoint a visiting staff, consisting of a limited number of physicians and surgeons, whose duty it should be to co-operate with one another and to suggest to the managers all items as to improvement in equipment.

Secondly, to see that the medical records of the hospital are kept up to a definite standard, and that nothing is omitted from the histories, operative findings, etc.

Thirdly, that these records shall be signed by the attending physician and filed by the hospital authorities. That they shall be the property of the hospital and open at all times to the inspection of the hospital authorities, and shall be kept by the hospital authorities as part of its property for purposes of reference.

Fourthly, that the operating room shall be open to a visit from any member of the staff or a committee of its members at any time, either during or between operations.

Objections might be raised to these regulations under the plea that they would interfere seriously with the liberty of the doctor and place him under suspicion. No man with a clear conscience need fear working in such an atmosphere. In all the large hospitals of the country the greatest publicity exists. The workings of institutions from the garret to the operating room are openly exposed, and the records are open to the inspection of all in authority. The operating rooms are always open and nothing transpires there that is not a matter of common knowledge. It has been urged that in dealing with private patients, that honor demands that their ailments should not become public property, and that what transpires is nobody's business but their own. Such specious arguments are of no weight. Even in a private house the ailments of the patient are private just so long as the rights of the community are respected in the matters of legality, decency and public health. When these are transgressed, the public demands information and protection. Impertinent perusals of the records from motives of curiosity can easily be guarded against by the appointment of a committee to guard them.

The most serious problem to meet is the exclusion of men from the institution who are considered unfit. After the open scrutiny of his work which would be possible under the system outlined above, a reasonably fair conclusion could be reached. On recommendation of the staff, it would be an easy matter for the hospital authorities to find some excuse for refusing to admit his patients.

---

MORPHINE HABIT.—Not one of the methods of cure involving the production of a period of unconsciousness, during which the final withdrawal is made, is safe.—*Medical World.*

